# Genomic Signatures for Sedimentary Microbial Utilization of Phytoplankton Detritus in a Fast-Flowing Estuary

**DOI:** 10.3389/fmicb.2019.02475

**Published:** 2019-11-05

**Authors:** Maria W. Smith, Lydie Herfort, Adam R. Rivers, Holly M. Simon

**Affiliations:** ^1^Center for Coastal Margin Observation & Prediction, Oregon Health & Science University, Portland, OR, United States; ^2^Institute of Environmental Health, Oregon Health & Science University, Portland, OR, United States; ^3^U.S. Department of Energy Joint Genome Institute, Walnut Creek, CA, United States

**Keywords:** metagenomics, microbial community, estuarine sediments, phytoplankton, Bacteroidetes, bacteriophage

## Abstract

In fast-flowing, river-dominated estuaries, “hotspots” of microbial biogeochemical cycling can be found within areas of extended water retention. Lateral bays located off of the North and South channels of the Columbia River estuary are proposed to be such hotspots. Previous metagenomic studies on water samples indicated that these regions function both as sources and sinks of biogenic particles, with potential to impact organic matter fluxes in the estuary. To extend this work, we analyzed 11 sediment metagenomes from three disparate bays: the freshwater Cathlamet Bay, and the brackish Youngs Bay and more saline Baker Bay located nearer the mouth to the south and north of the main channel, respectively. Samples were collected from upper layers of sediments in August of 2011 and 2013 for DNA extraction and metagenome sequencing. All metagenomes were dominated by bacterial sequences, although diatom sequences as high as 26% of the total annotated sequences were observed in the higher salinity samples. Unsupervised 2D hierarchical clustering analysis resulted in the eleven metagenome samples clustered into four groups by microbial taxonomic composition, with Bacteroides, diatom, and phage levels driving most of the grouping. Results of functional gene clustering further indicated that diatom bloom degradation stage (early vs. late) was an important factor. While the Flavobacteriia and Cytophagia classes were well represented in metagenomes containing abundant diatoms, taxa from the Bacteroidia class, along with certain members of the Sphingobacteriia class, were particularly abundant in metagenomes representing later stages of diatom decomposition. In contrast, the sediment metagenomes with low relative abundance of diatom and Bacteroidetes sequences appeared to have a metabolic potential biased toward microbial growth under nutrient limitation. While differences in water salinity clearly also influenced the microbial community composition and metabolic potential, our results highlight a central role for allochthonous labile organic matter (i.e., diatom detritus), in shaping bacterial taxonomic and functional properties in the Columbia River estuary lateral bay sediments. These results suggest that in fast-flowing, river-dominated estuaries, sediment microbial communities in areas of extended water retention, such as the lateral bays, may contribute disproportionately to estuarine organic matter degradation and recycling.

## Introduction

The role of estuaries in the global decomposition of organic carbon to CO_2_ and its loss to the atmosphere is a matter of much scientific debate (Cai, 2011). Estuaries dominated by fluvial processes, in particular, are thought to function largely as conduits promoting the flow of dissolved and particulate organic matter from the land to the coastal ocean. The fast-flowing Columbia River estuary experiences residence times of only a few days (Neal, 1972). This is due to powerful physical forcing that results in low mean water mass ages (mostly < 40 h) calculated for the mainstem estuary (Kärnä and Baptista, 2016) and short water transit times (Battin et al., 2008; Chawla et al., 2008). Such river-dominated estuaries may not be as effective as those with longer residence times in filtering out nutrients, organic matter and trace metals as water makes its way to the coast (Caffrey, 1995; van Beusekom and Brockmann, 1998; Small and Prahl, 2004; Garnier et al., 2010). Bruland et al. (2008) found support for this idea by examining tidal and river discharge influences on near-field plume chemistry. Nitrate, phosphate and silicic acid all displayed conservative mixing behavior in the Columbia River estuary, which was interpreted as a relative absence of biological activity. However, attempts to quantify nutrient transformations over the longer term encounter significant temporal and spatial complexity (Gilbert et al., 2013). At the same time other studies have, in contrast, uncovered evidence for an active heterotrophic community and biogeochemical transformations of organic matter in the estuary (Crump and Baross, 1996; Small and Prahl, 2004; Gilbert et al., 2013). Rates of bacterial production were found to be particularly high in the estuarine turbidity maxima (ETM), which are particle-suspension/retention zones that form near the leading edge of the incoming salt wedge (Jay et al., 2007; Simon et al., 2014). Enhanced microbial remineralization also occurs within several shallow, less well-studied lateral bay/tidal mudflat regions located both to the north and south of the main channels (Gilbert et al., 2013), wherein water age has been estimated up to 120 h under low flow and neap tide conditions (Kärnä and Baptista, 2016). Thus, it follows that even in river-dominated estuaries, estuarine sediments may provide a stable environment for development of microbial communities mediating biogeochemical cycling, particularly at the periphery of the main estuarine channels and in the associated lateral bays, where water residence time is lengthened.

Metagenome studies of estuarine microbial communities have focused mainly on the water column, with only a few analyzing bottom sediments [e.g., Pearl River, White Oak River (Jiang et al., 2009; Baker et al., 2015)], and Columbia River (Smith et al., 2015). These studies found genomic evidence for the involvement of estuarine sediment microorganisms in many essential biogeochemical processes including but not limited to organic matter consumption, methane production and turnover, and nitrogen, sulfur, and iron transformations (Jiang et al., 2009; Baker et al., 2015; Smith et al., 2015; Youngblut et al., 2015). Analysis of a temperate estuary of the White Oak River (North Carolina) indicated high diversity and heterogeneity of the microbial community and the prevalence of uncultured and poorly characterized taxonomic lineages (Baker et al., 2015). In a pilot study carried out on three sediment metagenomes collected in 2011 from the lower Columbia River estuary, Smith et al. (2015) discovered an apparent association of Bacteriodetes taxa with phytoplankton (diatom) detritus in estuarine sediments. Bacteroidetes taxa also appeared to have similar roles in the Columbia River water column (Smith et al., 2017). However, limitations in taxonomic resolution of sequence annotations made it unclear whether or how much the same taxa overlap in both habitats.

**FIGURE 1:**
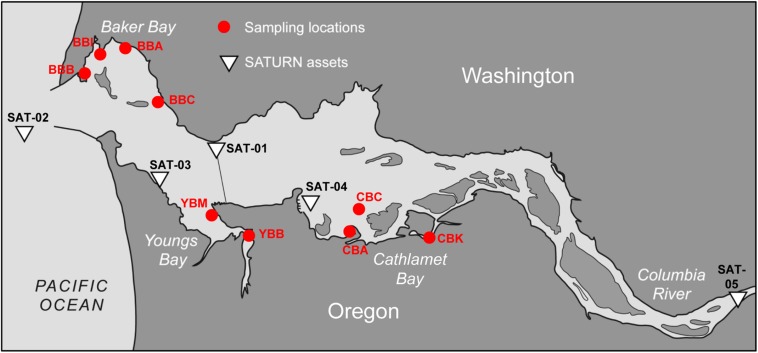
Contour map of the lower Columbia River estuary. The locations of nine sediment sampling sites are shown as red circles. The sampling site names are as the following: BBB, Baker Bay boat launch; BBI, Baker Bay Ilwaco Harbor; BBC, Baker Bay Chinook; BBA, Baker Bay airport; YBM, Youngs Bay mouth; YBB, Youngs Bay back; CBA, Cathlamet Bay site A; CBC, Cathlamet Bay site C; CBK, Cathlamet Bay Knappa. The white triangles show locations of the SATURN monitoring stations providing continuous flow-through physical and biogeochemical data. This figure was modified from Figure 1 of Baptista et al. (2015). © Higher Education Press and Springer-Verlag Berlin Heidelberg 2015.

In order to better understand the functional roles of heterotrophic microbiota in organic matter and nutrient utilization in the estuary, we analyzed metagenomes of eight sediment samples collected in August 2013 from three lateral bays (Baker, Youngs and Cathlamet Bays) of the lower Columbia River estuary (Figure 1). The eight-metagenome set was combined with the three sediment metagenomes from our pilot study (Smith et al., 2015), which were re-analyzed together with the new samples. The bays, which are shallow intertidal inlets, are connected either to the main North (Baker Bay) or South (Youngs and Cathlamet Bays) Channels (Figure 1). The sampling locations represented sites differing in a number of relevant properties, including sediment composition (Table 1), freshwater influence, water circulation, degree of tidal forcing and salt-water intrusion. Prior analyses of the diatom flora in estuarine sediments and the water column (Simenstad et al., 1984) indicated that (i) Baker Bay sites encountered the highest salinities of the three bays; (ii) the intertidal regions of Cathlamet Bay and Youngs Bay are mostly freshwater; and (iii) despite the influence of brackish and oceanic water near the mouth of Youngs Bay, diatoms found there are nevertheless most similar to those collected from freshwater regions (e.g., Cathlamet Bay). The mean surface salinity values recorded in the bays under high- and low-flow conditions, respectively, were Baker: 5.0, 16.8; Youngs: 0.1, 5.0 and Cathlamet: 0.0, 0.5 (Jay, 1984). There are significant freshwater inputs into Youngs and Cathlamet Bays that do not occur in Baker Bay. Thus, we hypothesized that the metagenome composition would be more similar between sediments of Youngs and Cathlamet Bays, and would differ from those in Baker Bay. While our results generally support this prediction, our analyses indicated that nutritional status, largely governed by the availability of labile diatom detritus, also had a dominant role in the structuring of sediment bacterial communities relative to either oceanic influences, or geographic location.

**TABLE 1:** Environmental metadata and metagenome statistics.

**Sample name**	**S.41_ BBB**	**S.184_ BBB**	**S.182_ BBI**	**S.186_ BBA**	**S.180_ BBC**	**S.43_ YBM**	**S.178_ YBM**	**S.42_ YBB**	**S.188_ CBA**	**S.190_ CBC**	**S.174_ CBK**
Lateral bay	Baker	Baker	Baker	Baker	Baker	Youngs	Youngs	Youngs	Cathlamet	Cathlamet	Cathlamet
Latitude	46.29	46.29	46.3	46.31	46.27	46.17	46.18	46.16	46.19	46.21	46.19
Longitude	−124.05	−124.05	−123.04	−124	−123.95	−123.85	−123.85	−123.81	−123.69	−123.69	−123.59
Collection Date	22-August-11	23-August-13	23-August-13	23-August-13	23-August-13	22-August-11	23-August-13	22-August-11	23-August-13	23-August-13	19-August-13
pH	7.5	7.2	7.5	7.7	7.5	6.6	7.2	6.3	6.9	6.7	7.4
% OM	1.8	2.9	0.7	0.5	0.6	5.4	1.1	8.8	2	2.3	0.7
NO3 (ppm)	8.1	5	3	3	3	3	3	2.5	6	4	3
NH4 (ppm)	2	28	14	13	15	150	8	170	44	22	14
Mn (ppm)	15.5	65	28	17	19	243	25	162	89	48	46
Fe (ppm)	20	159	70	60	45	214	131	254	140	97	75

Taxon_oid^a^	3300005832	3300005825	3300005828	3300005826	3300005824	3300005831	3300005830	3300005836	3300005827	3300005829	3300005833
Genome Size (Mbp)	1,382	529	786	671	520	1,099	934	1,845	640	880	1,210
CDS Count (million)	2.3	1.1	1.5	1.2	1.1	2.0	1.9	3.2	1.4	1.7	2.4
Average gene size (bp)	591	467	503	541	492	548	490	576	467	501	508
RNA%	0.75	0.95	0.67	0.59	0.72	0.84	0.9	0.78	1.07	0.77	0.78
Functional annotation (%)	48.54	43.53	32.54	28.6	35.11	45.32	40.99	49.15	46.78	48.37	50.77
Taxonomic annotation (%)	74.02	72.73	57.21	50.67	61.46	74.27	66.51	76.51	77.13	77.63	80.05
EGS (Mb)^b^	3.23	2.55	2.29	2.35	2.35	2.94	2.94	3.54	2.47	3.09	3.15
#EGS/metagenome	292	139	146	94	107	268	192	383	193	212	298
Archaea^c^	6.47	4.61	5.08	1.66	2.53	0.85	2.64	2.12	0.81	2.34	1.37
Bacteria^c^	92.92	93.01	74.8	65.73	78.97	97.68	91.71	96.99	97.52	96.69	97.82
Viruses^c^	0.08	0.58	0.64	0.52	0.48	0.16	0.18	0.05	0.38	0.05	0.07
Bacillariophyta^c^	0.01	0.56	15.49	26.06	14.78	0.15	3.1	0.01	0.22	0.18	0.28
Other Eukaryota^c^	0.53	1.25	3.98	6.04	3.24	1.16	2.37	0.84	1.06	0.73	0.46
Bacteroidia^d^	1.53	2.27	0.84	0.88	3.71	17.7	0.75	0.52	4.14	0.53	0.45
Cytophagia^d^	1.9	6.78	8.17	3.6	6.97	10.26	2.42	2.74	5.35	2.23	1.24
Flavobacteriia^d^	1.3	6.17	8.05	3.32	9.23	8.6	1.35	1.33	2.93	1.2	0.83
Sphingobacteriia^d^	1.08	2.04	2.16	1.04	2.43	7.96	1.22	3.61	4.22	2.96	2.48
Total Bacteroidetes^d^	6.12	17.52	19.49	9.02	22.5	44.81	6.16	8.55	16.84	7.28	5.27

*^*a*^Metagenome accession numbers in the IMG/M-ER of the DOE JGI, https://img.jgi.doe.gov/cgi-bin/mer/main.cgi. ^*b*^Effective bacterial genome size. ^*c*^Percentage of the total number of CDS with taxonomic annotations ≥ 30% identity ≥ 70% of the alignment length. ^*d*^Percentage of the sum of CDS annotated as Archaea, Bacteria and Viruses (total ABV).*

## Materials and Methods

### Sample Collection and Characterization

Sediment samples were collected from three lateral bays of the lower Columbia River estuary in August of 2011 and 2013. A total of 11 sediment samples were manually collected near shore during low tide, three on August 22nd, 2011 (Smith et al., 2015), and eight on August 23rd, 2013. Four sites were located in Baker Bay (designated BBA, BBB, BBC and BBI for different regions in the bay), two in Youngs Bay (YBM and YBB near the mouth and back regions, respectively) and three in Cathlamet Bay (designated CBA, CBC and CBK for different regions in the bay). Locations of the sample collection sites are shown on the map in Figure 1.

Sampling was carried out during typical late summer conditions. These are described as: (i) low river discharge with a relatively small plume in the coastal ocean, (ii) stratified estuarine water column, (iii) frequent and prolonged nearshore upwelling events inducing phytoplankton blooms in the coastal ocean; and (iv) large tidal intrusions transporting high-salinity and nutrient-rich oceanic water containing phytoplankton into the lower estuary and lateral bays adjacent to the river mouth (Jay, 1984; Small et al., 1990; Hickey and Banas, 2003; Roegner et al., 2011). Cores were collected from the sub-surface sediment (upper 1–10 cm) using sterile 50 mL Corning tubes, mixed and aliquoted in 500 mg portions. Most samples were collected from the shore at low tide, except for two locations in Cathlamet Bay (CBC and CBA) where the sampling was done during mid-flood tide by diving in the water from the R/V CORIE. Total DNA isolation was performed using two aliquots stored on dry ice or at −80°C.

### Biogeochemical Data

**FIGURE 2:**
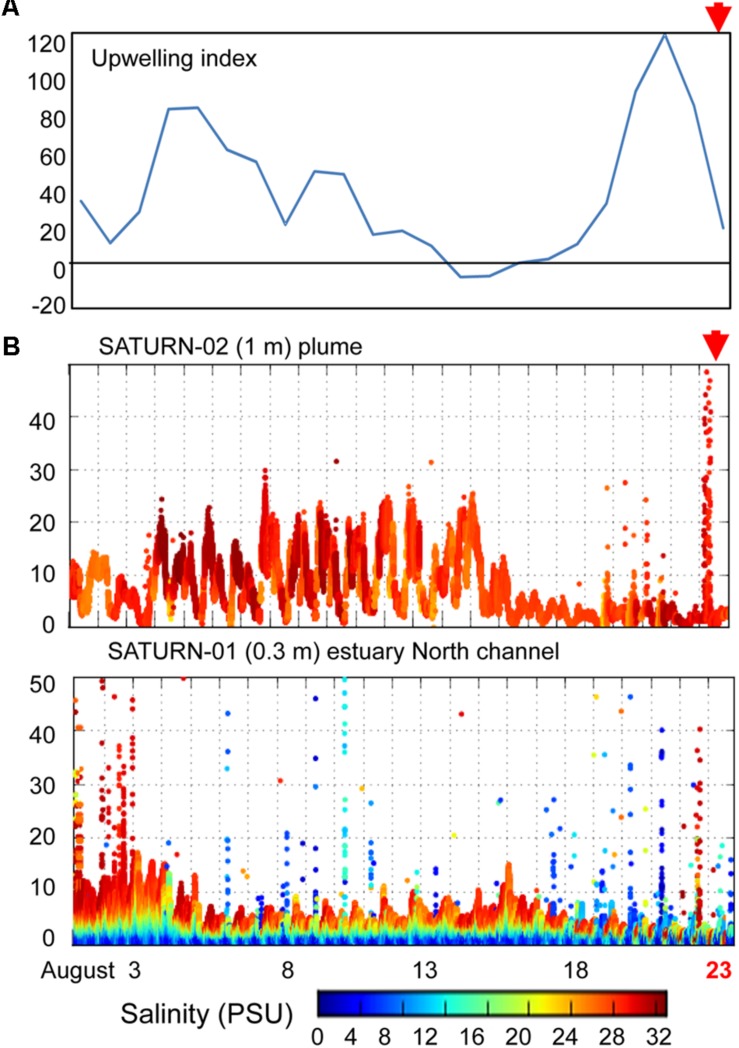
**(A)** A plot of daily calculations of the Coastal Upwelling Index (at 45° North). Values above zero indicate coastal upwelling. **(B)** Continuous flow-through measurements of chlorophyll *a* (chl *a*) concentrations (*Y*-axis, μg/L) at SATURN endurance stations from August 1 to August 23, 2013 (*X*-axis, time in days). Chl *a* values are colored by water salinity (the color scale is shown at the bottom). The presented chl *a* data have not been cross-calibrated using laboratory measurements in collected water samples, thus, they cannot be directly compared between sensors, however, we used them to examine the temporal trends at each station. Red arrows indicate time of sampling.

Near real-time continuous sensor measurements of salinity and chlorophyll *a* (chl *a*) concentrations were collected by observation stations of the Center for Coastal Margin Observation & Prediction (CMOP) Science and Technology University Research Network (SATURN) (Baptista et al., 2015). The station locations are shown in Figure 1. River discharge and water temperatures were similar in 2011 and 2013 around times of sediment collection. Data from SATURN-01 and -02 stations, corresponding to sediment sampling times, are shown in Figure 2. These and other relevant physical and biogeochemical data are also available through the CMOP website^1^ (Baptista et al., 2015). The bulk chemical properties of the collected sediment cores (Table 1) were analyzed by Agri-Check (Umatilla, OR, United States) from frozen (−20°C) aliquots.

### Detection of Phytoplankton From Continuous Water Sensor Data

We examined SATURN chl *a* and salinity sensor data to determine phytoplankton fluxes into the estuary from the ocean and/or river close in time to sediment sample collections (Figure 2). Upwelling conditions were observed from late June to August of 2011 in the coastal ocean adjacent to the Columbia River mouth and by estuarine water sensors (SATURN-03 station) near Youngs Bay. Sensor data showed a clear association between elevated chl *a* concentrations and high salinity water at all three measured depths (2.4, 8.2, and 13 m) (Smith et al., 2015). Upwelling was also observed in 2013 (Figure 2A), but was more discontinuous, with a downwelling period (14–17 August) followed by a strong upwelling event at the end of August, near the time of sediment collections. This relatively strong upwelling event in late August 2013 was associated with a large coastal bloom, resulting in chl *a* concentrations in the Columbia River plume (SATURN-02 station) reaching 50 μg/L (Figure 2B). The SATURN-01 station, located in the North Channel at the eastern border of Baker Bay (Figure 1), recorded several chl *a* peaks in the second half of August with concentrations reaching as high as 40 μg/L. These peaks were associated with both higher (24–30 PSU) and lower (0–12 PSU) salinity water (Figure 2B).

### DNA Preparation and Sequencing

Genomic DNA was extracted from ∼1 g sediment using the Fast DNA spin kit for soil (Thermo Fisher Scientific, Waltham, MA, United States). DNA concentrations were quantified using the PicoGreen dye (Invitrogen Corp., Carlsbad, CA, United States), on a ND-3300 Nanodrop fluorospectrometer (Thermo Scientific, Wilmington, DE, United States). Approximately 10–30 μg of total DNA was generated from each sample. DNA library preparation and sequencing were performed at the Oregon Health and Science University Massively Parallel Sequencing Shared Resource (OHSU-MPSSR). In total, 11 libraries, each containing DNA from a single sediment sample, were analyzed with Illumina HiSeq 2000 Sequencer (Illumina, San Diego, CA, United States), using 2 × 100 bp paired-end sequencing and the standard base-calling pipeline (Illumina Pipeline-0.3). Sequencing statistics are listed in Table 1.

### Metagenome Assembly and Analysis

Three metagenomes from samples collected in 2011, namely, S.41_BBB, S.42_YBB, and S.43_YBM, were originally analyzed as described in Smith et al. (2015) (with the following taxon object IDs: 3300001371, 3300001372, and 3300001373, respectively). However, both reference databases and genome analysis tools have undergone significant change since that time. Thus, to enable direct comparisons with the 8 new sediment metagenomes generated from samples collected in 2013, the 2011 metagenomes were re-assembled following standard protocols at the DOE Joint Genome Institute (JGI), CA, United States and re-analyzed alongside the new 2013 metagenomes in the following manner. The 100 bp paired-end samples were first quality controlled using BBqc version 35.08, a program in the BBtools suite^2^. BBqc trims Illumina adaptors, artifact sequences, quality-trims the reads then maps the reads to the human genome to remove any human contamination. For each metagenome, the quality-trimmed, paired-end reads were merged with BBmerge version 8.0 (Bushnell et al., 2017). Metagenomes were assembled individually and the resulting 2.1 billion unmerged reads and 530 million merged reads were assembled on a 32 core, 1TB memory node using Megahit version 0.3.0-beta (Li et al., 2015). Megahit used the paired-end information to iteratively assemble contigs using kmer values from 31 to 121 in increments of 10. Overall, 13 GB of DNA was assembled into 9.1 million contigs with half of the assembled data in contigs with a length greater than 1256 bp (N50).

Subsequent analysis of the metagenomes was done using the Integrated Microbial Genomes with Microbiome Samples - Expert Review web server (IMG/M-ER of the DOE JGI)^3^ (Markowitz et al., 2012). The IMG/M-ER server performed prediction of the most probable RNA coding genes, peptide coding sequences (CDS), and subsequent functional and taxonomic annotations (Markowitz et al., 2012). Taxonomic analyses were first done at the domain level using a cutoff of ≥30% predicted amino acid sequence identity over ≥70% of the length of a pair-wise alignment for a given gene with the corresponding top hit reference. The relative abundance of a taxon was calculated in a metagenome as the sum of all corresponding genes divided by the sum total of all annotated CDS (Table 1). The additional cutoff ≥60% identity (over ≥ 70% of the amino acid alignment length) provided family to genus level identifications (Konstantinidis and Tiedje, 2005).

We compared the old (IDBA-UD assembly, 2013 analysis, Smith et al., 2015) and new (MegaHit assembly, 2015 analysis) taxonomic annotations of the S.41_BBB, S.42_YBB, and S.43_YBM metagenomes. The new annotations were based on a significantly enlarged reference database and contained additional novel phylogenetic categories at all taxonomic levels. This improved the proportion of taxonomically annotated CDS in the new annotations to 74–78% of the total CDS counts (Table 1), compared to 66–70% in the earlier analysis (Smith et al., 2015). Comparison of the categories present across both annotations produced correlation coefficients of 0.96–0.99.

Functional comparison of metagenomes was performed by normalization to the effective bacterial genome size (EGS) (Table 1), as described previously (Raes et al., 2007; Smith et al., 2013). For all four types of functional annotations analyzed (COG, PF, Enzyme, and KO), the relative abundance value for a gene category in a metagenome was calculated as the number of corresponding predicted genes (CDS) divided by the number of effective bacterial genomes in this metagenome. The resulting value is designated as the number of genes per effective bacterial genome (‘genes/genome’ in the figures).

Table 1 shows large variation in the presence of Bacillariophyta (diatom) sequences among the sediment metagenomes. To prevent bias from this uneven distribution of diatom sequences, all eukaryotic sequences were excluded from calculations, and the relative abundance of a specific archaeal, bacterial or viral taxon in a given metagenome was calculated as the percentage of corresponding genes divided into the sum total of all genes annotated as Archaea, Bacteria, and Viruses at ≥30% identity level (‘total ABV’) (Table 1). Note that relative abundance calculations corresponded solely to those genes that were sequenced at sufficient depth for assembly.

### Inferring Physiological State of Phytoplankton From Gene Ratios

We used a strategy based on diatom protein-coding genes, of which >11,000 are nuclear-encoded and <200 (<2% of the total) are chloroplast-encoded (Armbrust et al., 2004; Lopez et al., 2005), to infer the general physiological state of phytoplankton present at the time of sampling. This approach relies on the fact that, upon cell death, diatom chloroplast DNA has double the membrane protection of nuclear DNA (4 membranes compared to 2) (Lang et al., 1998). We calculated the percentage of the chloroplast-encoded RuBisCO (EC 4.1.1.39) sequences (‘% RuBisCO’) relative to all other identified diatom sequences that have functional annotations based on the ENZYME (EC) database. The analysis was performed only for the metagenomes that contained ≥ 10 diatom RuBisCO genes.

### Unsupervised 2D Clustering and Correlation Analyses

Unsupervised 2D hierarchical clustering (Spearman Rank correlation and average linkage) of the normalized relative abundance of taxonomic and functional gene categories was performed using software programs Cluster and TreeView (Eisen et al., 1998). Taxonomic (expressed as percentages of the total) and functional (expressed as numbers of genes per effective bacterial genome) genes were grouped by similarity and gene abundance patterns were revealed across the 11 metagenomes. Correlation analyses of the abundance values (functional or taxonomic categories separately) was performed in Excel V14.7.3 in the following manner: (1) Highly abundant gene(s) or taxa were observed within a cluster; (2) Corresponding gene(s) were designated as a ‘query;’ and (3) Correlation analysis was run for the query against the genes or taxa in all of the clusters (the others in addition to the one it was identified in) by calculating pair-wise correlation (Spearman rank) coefficients and significance (Zar, 1972). This approach allowed identification of the taxa and functional genes that showed the abundance patterns most similar to the query. The selected categories were further visualized in Excel V14.7.3 by plotting (bubble plots and bar graph diagrams).

To test for evidence of significant internal structure, similarity profile analysis (SIMPROF; Clarke et al., 2008) was also performed using The Plymouth Routines In Multivariate Ecological Research (PRIMER) software v.6 (PRIMER-E Ltd, United Kingdom). This analysis was carried out on dendrograms of the hierarchical clusters using a resemblance matrix of Spearman Rank correlations (average linkage) computed from the relative abundance of genes.

### Availability of Data and Materials

The 11 assembled Illumina datasets are available in the IMG/M-ER metagenome database, designated with GOLD ID Gs0047387 (Marine and estuarine microbial communities from Columbia River Coastal Margin), including corresponding metadata. Table 1 shows the IMG/M-ER accession numbers: taxon_oid (taxon object identification) for each metagenome.

## Results

### Biogeochemical Characterization of Sediment Samples

Sediments were collected from 9 different locations in Baker, Youngs and Cathlamet Bays of the lower Columbia River estuary (Figure 1). Geochemical data (Table 1) varied widely between sites and samples, with a large range of values measured for organic matter content (0.5–9%) and concentrations of ammonia (2–170 ppm), iron (20–254 ppm), and manganese (16–162 ppm). Youngs Bay sediment samples had the highest proportion of organic matter (on average, 5.1 ± 3.8% vs. 1.3 ± 1.0% and 1.7 ± 0.85% in Baker and Cathlamet Bay sediments, respectively), and the highest concentrations of ammonia, iron, and manganese. In contrast, nitrate content was generally lower in Youngs Bay and higher in the other two bay sediments (Table 1).

### Metagenome Features and Domain Composition

The sediment metagenome assemblies ranged in size from 0.5 to 1.8 Gbp (Table 1). In total, the 11 metagenome assemblies contained 10.5 Gbp of sequence information. From 1.1 to 3.2 million CDS were predicted for each individual metagenome (Table 1), with a combined total of approximately 20 million identified genes. The median predicted gene size was 517 bp. During downstream analysis, each predicted gene of an assembled scaffold was annotated separately and considered as a single independent entry. Approximately 1% of predicted genes corresponded to non-coding RNA (including rRNA and tRNA). Approximately 70 and 43% of all predicted protein-coding genes were associated with taxonomic and/or functional annotations, respectively (Table 1). The combined average effective genome size (EGS) was 2.8 Mbp. This average is likely an underestimation, since the calculation included only annotated bacterial peptides (Raes et al., 2007; Konstantinidis et al., 2009). The EGS values were used to predict corresponding genome equivalents in the individual metagenomes (ranging from 94 to 383 with a mean of 211, Table 1), and to normalize across metagenomes.

All 11 sediment metagenomes were dominated by Bacteria, with 66–98% of all predicted protein-coding genes annotated as corresponding to this domain (Table 1). The lowest relative abundance of bacterial genes was observed in three of the 2013 metagenomes from Baker Bay, due to the presence of Bacillariophyta (diatom) sequences (comprising 15–26% of the total annotated genes). In the other sediment metagenomes, neither diatom nor other eukaryotic sequences were generally abundant, accounting for <2% of the total annotated genes (Table 1).

### Archaeal Community Composition

Genes annotated as Archaea were enriched to >4% of the total ABV (Archaea, Bacteria, and Viruses) in the three westernmost Baker Bay metagenomes (S.41_BBB, S.184_BBB, S.182_BBI) when compared to others (0.8–2.6%) (Table 1). We normalized gene abundances using two different approaches (percentages of the total ABV and genes per genome) and directly compared them for: (i) all predicted archaeal taxonomic gene annotations (Table 1); and (ii) all predicted functional gene annotations of the KO:K04479 category encoding the archaea-specific housekeeping enzyme DinB-like DNA polymerase IV. Both categories displayed similar abundance patterns across the 11 metagenomes, with a correlation coefficient of 0.95, indicating good consistency between the two approaches.

**FIGURE 3:**
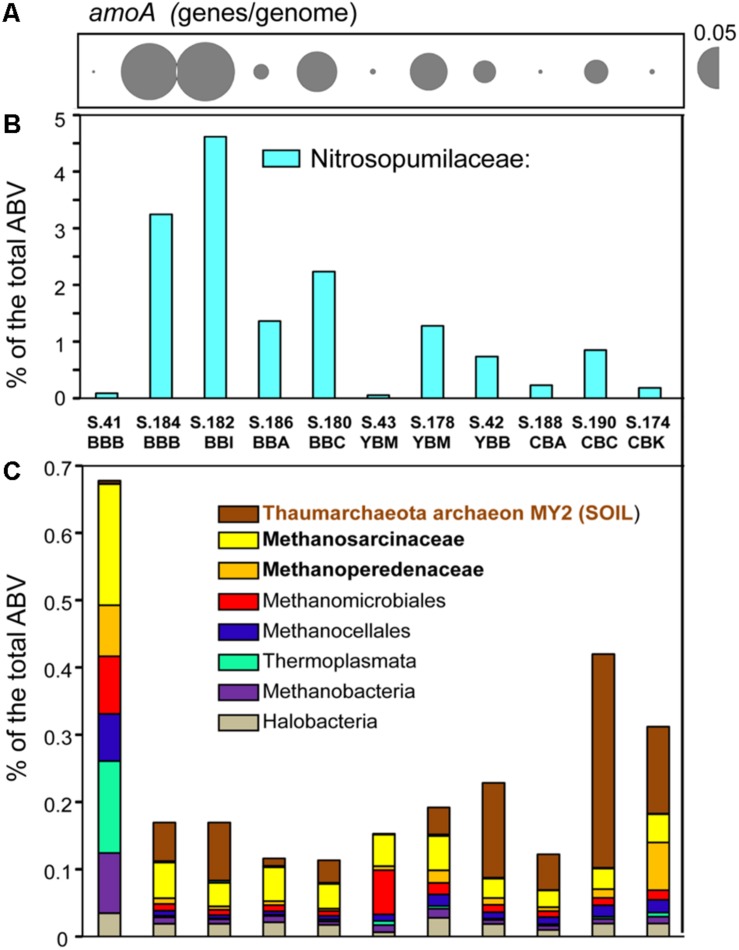
Assessment of the archaeal community composition and metabolic potential using the abundance of **(A)** the functional gene category of archaeal ammonia monooxygenase subunit A (*amoA*) (Pfam12942) with the values expressed as the number of genes per genome; **(B)** the Nitrosopumilaceae family; and **(C)** all other archaeal taxa (with the three most abundant taxa shown in bold lettering), and values expressed as the percentages of the corresponding genes relative to total predicted archaeal, bacterial and viral genes (% total ABV). The predicted taxonomic gene annotations were based on ≥60% identity over ≥70% of the alignment length. The abundance values for *amoA* are proportionate to the bubble width, and the scale is indicated with a half-bubble to the right with the genes/genome value indicated by a number. Metagenome names are composed of the sample name (BBB, Baker Bay boat launch; BBI, Baker Bay Ilwaco Harbor; BBC, Baker Bay Chinook; BBA, Baker Bay airport; YBM, Youngs Bay mouth, YBB, Youngs Bay back; CBA, Cathlamet Bay site A; CBC, Cathlamet Bay site C; CBK, Cathlamet Bay Knappa) and the database sample number.

As reported previously for archaea in metagenomes from the Columbia River estuarine water column and sediments (Smith et al., 2013, 2015), the Thaumarchaeota were dominant across the 11 sediment samples. The highest abundances were observed for sequences representing the family Nitrosopumilaceae. These were enriched (1.5–4.5% of the total ABV) in the metagenomes of the four Baker Bay and the Young Bay Mouth (S.178_YBM) samples collected in 2013, and were also generally present in the majority of sediment metagenomes at lower relative abundance (<1%) (Figure 3). Additional analysis indicated that the closest sequenced relative to the Nitrosopumilaceae taxa was the marine ammonia-oxidizing thaumarchaeon “*Candidatus* Nitrosopumilus sediminis” AR2 from arctic marine sediment (Park et al., 2012). We also analyzed predicted *amoA* genes, encoding ammonia monooxygenase subunit A (PF12942), a marker for archaeal ammonia oxidation (Figure 3A). The abundance patterns of *amoA* and the Nitrosopumilaceae family were highly correlated (*p* ≤ 0.001) across the sediment metagenomes (Figures 3A,B), suggesting a potential role for these archaea in estuarine nitrogen cycling. However, the abundance values were not correlated with the total ammonia concentrations measured in the sediment samples (data not shown).

Another relatively abundant Thaumarchaeota taxon present (up to 0.5% of all sequences) was closely affiliated with the environmental isolate archaeon MY2 originally found in a deep oligotrophic soil horizon (Jung et al., 2014). This sequence was found enriched in the sediments of Youngs Bay back and Cathlamet Bay (Figure 3C), which are mainly freshwater sites with little exposure to salinity intrusions from the ocean endmember. Other archaeal sequences were affiliated with Euryarchaeota (mostly methanogens), including the families Methanosarcinaceae and Methanoperedenaceae (Youngblut et al., 2015), and the order Methanomicrobiales. These taxa were highly enriched in S.41_BBB, as described (Smith et al., 2015), and relatively uniformly distributed in the other 10 sediment metagenomes, albeit at lower abundance (Figure 3, note difference in scale between Figure 3B and Figure 3C).

### Bacterial Community Composition

**FIGURE 4:**
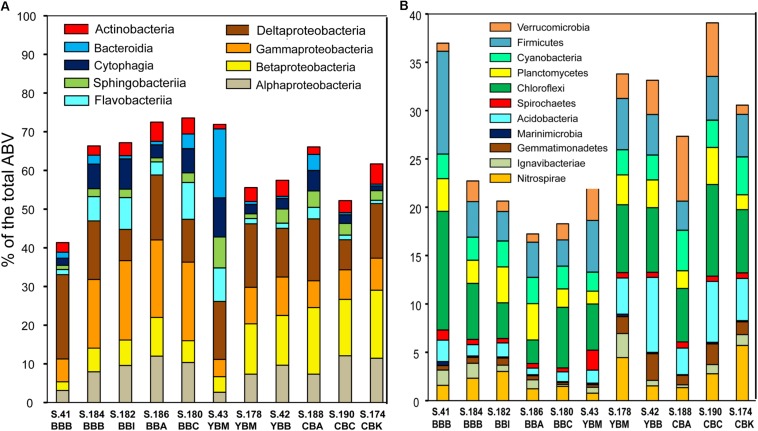
Taxonomic composition of Bacteria from predicted gene annotations in the assembled sediment metagenomes. Predicted peptides were annotated using the cutoff of ≥30% identity over ≥70% of the alignment length. Relative taxon abundances are expressed as percentages of the corresponding genes to total predicted archaeal, bacterial and viral genes (% total ABV). **(A)** Classes from the top two most abundant bacterial phyla, Proteobacteria and Bacteroidetes; **(B)** next 11 most abundant bacterial phyla. Metagenome names are described in Figure 3.

The metagenomes were dominated by three bacterial phyla: Proteobacteria, Bacteroidetes (all four classes: Bacteroidia, Cytophagia, Sphingobacteriia, and Flavobacteriia), and Actinobacteria (Figure 4A). On average, these phyla corresponded to up to 65% of all annotated genes in the sediment (Figure 4A). In addition, 11 other bacterial phyla showed high relative abundance, representing >2% of all taxonomically annotated sequences in at least one sediment metagenome (Figure 4B). The lowest abundance of bacterial genes and genome equivalents was observed from sediments collected in Baker Bay. This appears to be due to the relatively high abundance of both diatom and archaeal sequences present in most of these samples (Table 1).

**FIGURE 5:**
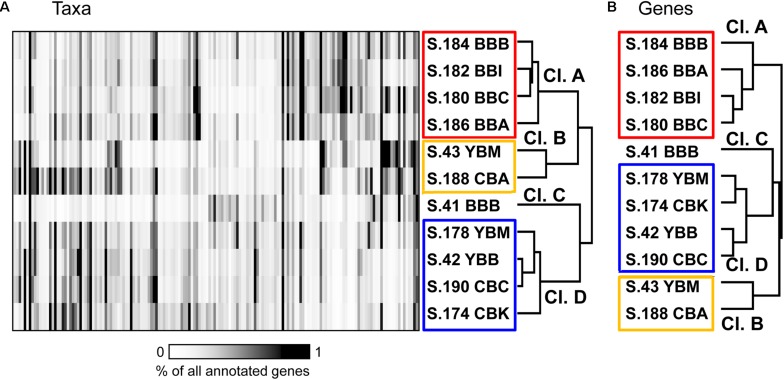
**(A)** 2D Hierarchical clustering (Spearman correlation, average linkage) of bacterial families (*X*-axis) in metagenomes (*Y*-axis). In total, 160 bacterial families were selected, each representing ≥0.35% of all annotated prokaryotic genes across metagenomes. For each family, abundance was calculated as the percentage of all corresponding genes (annotated using ≥60% sequence identity over >70% of the alignment length) to total annotated bacterial, archaeal and viral genes in the corresponding metagenome. Abundance values are shaded from white to black on a scale indicating percentages from low to high, respectively, as shown on the scale below the plot. **(B)** Functional gene categories (pfam), with the abundances expressed as numbers of genes per effective genome equivalent (genes/genome). One thousand three hundred and eighty-three genes were selected as having average abundance ≥1 gene/genome. Metagenome names are described in Figure 3.

Bacterial community composition was compared across metagenomes at the family/genus level (≥60% identity over ≥70% of the alignment length; Konstantinidis and Tiedje, 2005). One hundred sixty of the most abundant bacterial taxa, each representing ≥0.35% of the total ABV (corresponding to >5000 genes total in all metagenomes), were selected for 2D hierarchical clustering analysis. Results showed four major metagenome clusters (Figure 5A). Cluster A contained all four 2013 Baker Bay samples (S.184 BBB, S.186 BBA, S.182 BBI, S.180 BBC). One Baker Bay metagenome, S.41_BBB collected in 2011, formed a cluster separate from all other metagenomes (cluster C). The other two clusters contained a mix of metagenomes collected in different years and bays: cluster B consisted of two samples, one from Youngs Bay (S.43 YBM, collected in 2011) and one from Cathlamet Bay (S.188 CBA, collected in 2013), and cluster D contained the remaining four metagenomes from Youngs and Cathlamet Bays (collected in both 2011 and 2013) (Figure 5A).

### Bacterial Taxa Associated With Cluster-Specific Enrichment Patterns

**FIGURE 6:**
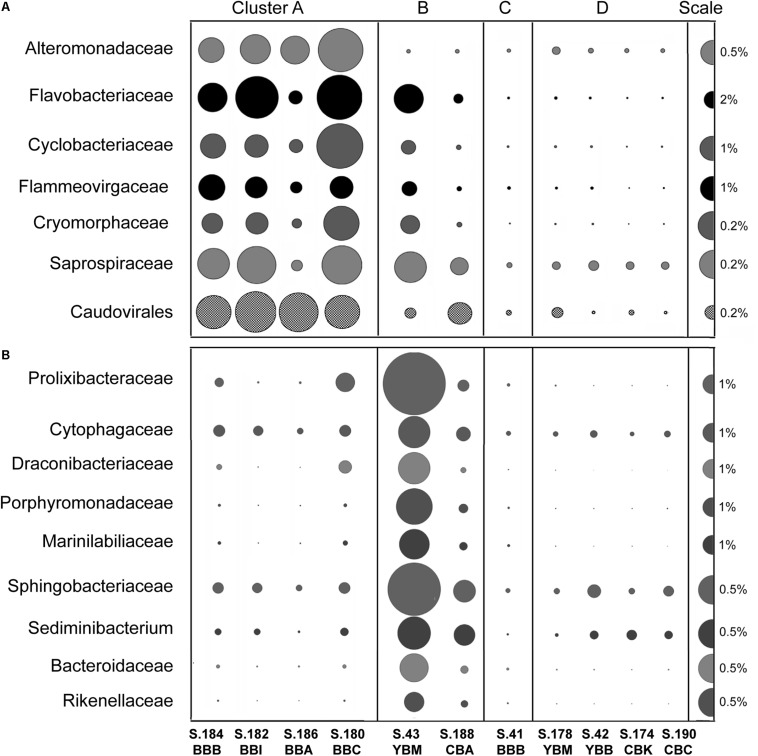
**(A)** Families of potential bloom-utilizing bacteria abundant in Cluster A metagenomes together with the bacteriophage order Caudovirales. **(B)** Bacteroidetes families/genera abundant in cluster B metagenomes (in S.43_YBM in particular). Abundance values are expressed as percentages of the corresponding genes relative to the total annotated archaeal, bacterial and viral genes in a given metagenome (% of total ABV). All annotations are based on ≥60% identity over ≥70% of the alignment length. The abundance values are proportionate to the bubble width. The scale for each row is indicated with a half-bubble to the right, and its diameter corresponds to a percentage value indicated by a number. The metagenomes are grouped into four clusters (A, B, C, and D) corresponding to those shown in Figure 5.

We examined the bacterial taxonomic clustering diagram (Figure 5A) to identify bacterial families and genera associated with the three major clusters (A, B, and D). Cluster A, composed of metagenomes collected in Baker Bay in 2013, had high relative abundance of taxa from the phylum Bacteroidetes (9–22% of Bacteroidetes, including Flavobacteriaceae, Cyclobacteriaceae, Sphingobacteriaceae), and several Gammaproteobacteria (Alteromonadaceae, marine gammaproteobacteria HTCC2143, 2148) (Figure 6A and Table 1). Three of the four metagenomes comprising cluster A also contained high relative abundance of Bacillariophyta sequences (15–26%, Table 1) corresponding to over a hundred thousand diatom genes in each metagenome). The fourth metagenome with a similar pattern of bacterial enrichment (S.184_BBB) contained only 0.56% diatom sequences (Figure 6A).

Cluster B metagenomes also showed dramatic enrichment of Bacteroidetes taxa (Figure 6B), 45 and 17% for S.43_YBM and S.188_CBA, respectively (Table 1). Some of the Bacteroidetes families in cluster B metagenomes overlapped with those enriched in cluster A (i.e., Saprospiraceae and Flavobacteriaceae in Figure 6A), while others were enriched exclusively in cluster B (Figure 6B). In contrast to cluster A, the relative abundance of the Gammaproteobacteria Alteromonadaceae was not elevated in cluster B (Figure 6A).

The metagenome S41_BBB in cluster C was most similar to Cluster D metagenomes (Figures 5, 6). S41_BBB contained relatively high abundance of sequences related to methylotrophic and syntrophic bacteria (generally representing sulfate reducers, mostly Deltaproteobacteria, and the Anaerolineaceae family of the phylum Chloroflexi, described in detail in Smith et al. (2015). Cluster D had high relative abundance of sequences representing both Proteobacteria and Acidobacteria, including a family of chemolithotrophic Betaproteobacteria (Hydrogenophilaceae; Orlygsson and Kristjansson, 2014) and two families of soil and rhizosphere associated bacteria: Acidobacteriaceae (Acidobacteria) and Bradyrhizobiaceae (Alphaproteobacteria).

### Bacterial Metabolic Potential

Two-dimensional hierarchical clustering analysis was also performed for functional gene annotations. General comparison of functional gene categories was performed using PF (pfam, protein families) annotations (Finn et al., 2016). Hierarchical clustering was performed with 1383 PF categories selected as having abundance ≥ 1 gene/genome in at least 2 metagenomes. Because the sediment communities were dominated by Bacteria, the majority of functional gene categories reflected bacterial metabolic potential. Although there were some topological differences between the clustering diagrams, the four major clusters observed at the functional level (Figure 5B), were the same as those found at the taxonomic level (Figure 5A). These patterns were determined to be significant by similarity profile analysis (Clarke et al., 2008). Analysis of the biogeochemical data, however, including organic matter content and nutrient concentrations found in Table 1, did not elucidate any significant correlations with the metagenome clustering patterns (data not shown).

### Differences in Transporter Gene Categories Across Clusters

**FIGURE 7:**
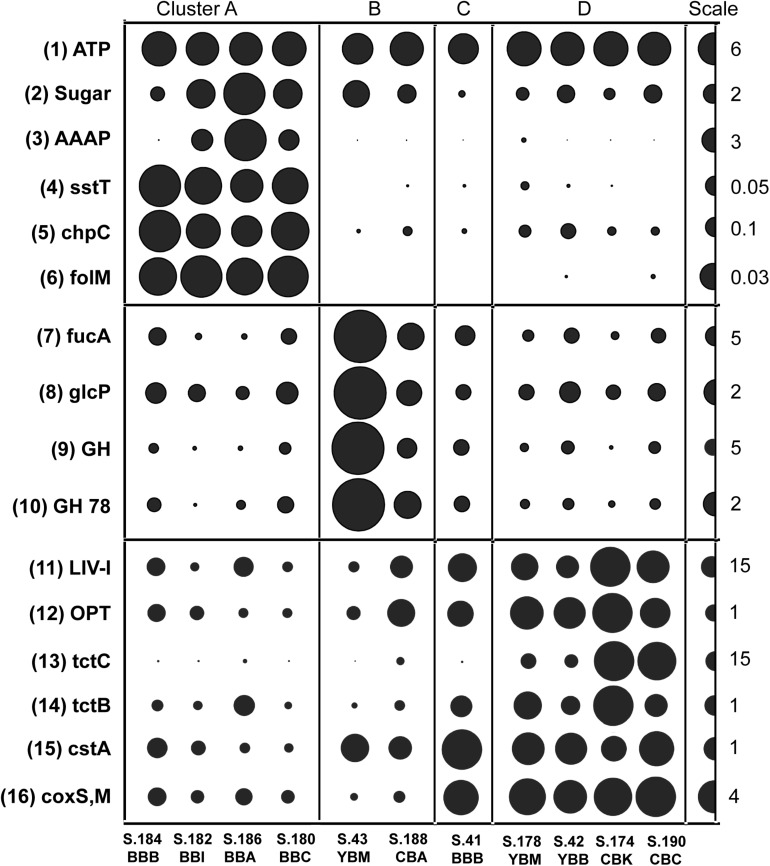
Functional gene categories enriched in particular metagenome clusters (genes/genome). The abundance values are proportionate to the bubble width. The scale for each row is indicated with a half-bubble to the right, and its diameter corresponds to genes/genome values shown with a number. The metagenomes are grouped into four clusters (A, B, C, and D) corresponding to those shown in Figure 5. The following gene categories are shown: (1) *ATP*, H(+)-transporting two-sector ATPase (EC:3.6.3.14); (2) Sugar, sugar transporters MFS (PF00083); (3) *AAAP*, transmembrane amino acid transporter protein (PF01490); (4) *sstT*, serine/threonine transporter (K07862); (5) *chpC*, chemosensory pili system protein (KO:K06598); (6) *folM*, dihydromonapterin reductase/dihydrofolate reductase (KO:K13938); (7) *fucA*, alpha-L-fucosidase (EC:3.2.1.51); (8) *glcP*, fucose permease (COG0738); (9) GH, glycosyl hydrolase families GH 49,9,43,2,65 (PF03718 + 14498 + 00759 + 04616 + 00703); (10) GH 78, bacterial alpha-L-rhamnosidase (PF05592); (11) *LIV-I*, branched-chain amino acid transporters (PF02653 + 12399); (12) *OPT*, oligopeptide transporter (PF03169); (13) *tctC*, tripartite tricarboxylate transporter family receptor (PF03401); (14) *tctB*, tripartite tricarboxylate transporter B (PF07331); (15) *cstA*, carbon starvation protein (PF02554 + 13722); (16) *coxS,M*, carbon-monoxide dehydrogenase (KO:K03518 + 3519).

Functional genes associated with the cluster-specific enrichment patterns were identified. These were compared generally to a positive control, the broad category of proton-transporting two-sector ATPases (EC:3.6.3.14), showing uniform distribution across the sediment metagenomes (Figure 7, top panel). Significant correlations (*p* ≤ 0.001) were uncovered in Cluster A for functional genes implicated in the uptake of fresh phytoplankton-derived labile dissolved organic carbon (DOC) (Poretsky et al., 2010), including transporters for sugars (MFS, PF00083), amino acids (AAAP, PF01490); serine/threonine (sstT, KO:K07862) (Figure 7), nicotinamide mononucleotide (PF04973), and nucleotide-sugar (PF04142).

All four cluster A metagenomes also showed dramatic enrichment of two functional gene categories potentially associated with particle colonization by free-living bacteria (Figure 7). The first category is involved in chemotaxis and encodes a chemosensory pili system protein, ChpC (KO:K06598) and the second category encodes dihydromonapterin reductase/dihydrofolate reductase, *folM* (KO:K13938).

Cluster B metagenomes contained a relative overabundance of functional gene categories involved in phytoplankton cell wall utilization, including sugar permeases and the CAZymes fucose permease glcP (COG0738), alpha-L-fucosidase fucA (EC:3.2.1.51), bacterial alpha-L-rhamnosidase GH 78 (PF05592), and glycosyl hydrolase families GH 49, 9, 43, 2, 65 (PF03718, 14498, 00759, 04616, 00703). All showed a similar abundance pattern and were subsequently combined within a single section in Figure 7 (middle panel). S.43_YBM and S.188_CBA contained approximately 3X and 2X more, respectively, of these sequences relative to the mean of the other metagenomes.

Cluster C and D metagenomes were enriched in bacteria carrying functional genes involved in recalcitrant DOC uptake (Figure 7, bottom panel). Gene categories identified included the branched-chain amino acid transporter *liv-I* (PF02653, 12399), oligopeptide transporter *opt* (PF03169), and the tripartite tricarboxylate transporter (*tct*) family receptors *tctC* (PF03401) and *tctB* (07331). In addition, cluster C and D metagenomes contained a relative overabundance of the broad functional gene category encoding carbon-monoxide dehydrogenase subunits of the CODH family (*coxS* and *coxM*, KO:K03518, 3519) (Figure 7). This category was significantly negatively correlated (*p* ≤ 0.001) with bloom-utilizing Bacteroidetes sequences, indicating that the decreased numbers of bacterial bloom-utilizers was associated with the enrichment of chemolithotrophic Bacteria and Archaea. Furthermore, several functional gene categories involved in nutrient scavenging (e.g., carbon starvation protein *CstA;* PF02554, 13722) (Figure 7) and an extracellular solute-binding protein (PF01547) were also enriched in cluster B, C, and D metagenomes (all containing low relative abundance of diatom genes), but not in cluster A metagenomes.

### Functional Markers for Biogeochemical Processes and Salinity Adaptation

**FIGURE 8:**
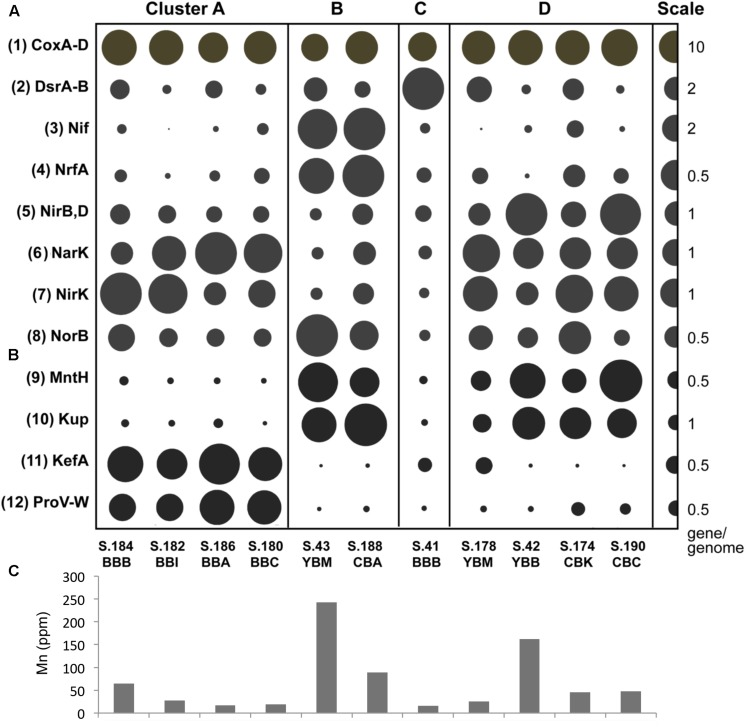
**(A)** Functional gene categories associated with sulfur and nitrogen cycle gene pathways, with abundance expressed as genes/genome. The abundance values are proportionate to the bubble width. The scale for each row is indicated with a half-bubble to the right, and its diameter corresponds to genes/genome values indicated by a number. The metagenomes are grouped in four clusters (A, B, C, and D) corresponding to those shown in Figure 5. The following gene categories are shown: (1) *cox*, cytochrome-c oxidase (EC:1.9.3.1), a gene involved in oxidative phosphorylation; (2) *dsr(A, B)*, dissimilatory sulfite reductases (desulfoviridins, COG2221); (3) *nif*, nitrogenase involved in nitrogen fixation (EC:1.18.6.1, K02585, K02586, K02587, K02588, K02591); (4) *nrfA*, nitrite reductase (cytochrome c-552, ammonia-forming) involved in dissimilatory nitrate reduction to ammonia (DNRA) (EC:1.7.2.2, KO:K03385); (5) *nirB* and *nirD*, nitrite reductases (NADH) involved in dissimilatory nitrate reduction (DNRA) (EC:1.7.1.15, K00362, K00363); (6) *narK*, nitrate/nitrite transporter involved in nitrate assimilation (KO:K02575); (7) *nirK*, nitrite reductase (NO-forming) involved in denitrification (EC:1.7.2.1, K00368); (8) *norB*, nitric-oxide reductase (cytochrome c) involved in denitrification (EC:1.7.2.5, K04561). **(B)** Functional genes associated with manganese and potassium transport and response to osmotic stress (genes/genome). The following gene categories are shown: (9) *mntH*, manganese transport protein (K03322); (10) *kup*, K+ uptake protein (K03549); (11) *kefA*, K+ efflux system protein (K05802); (12) *proV-W*, glycine betaine/proline transport system (K02000-2001). **(C)** Mn concentrations from bulk chemistry measurements of sediment samples. The sediments correspond to the metagenome labels shown above the bars **(B)**.

Further detailed examination of the functional gene clustering diagram revealed differential enrichment of genes representing microbial pathways involved in sediment biogeochemical processes and salinity adaptation (Figure 8). These genes, described below, are contrasted with the gene category *cox* (EC:1.9.3.1), encoding multiple components of cytochrome-c oxidase (the key enzyme involved in oxidative phosphorylation), which showed a relatively uniform enrichment pattern across all of the sediment metagenomes (Figure 8A, top).

Enrichment patterns for gene markers of sulfur, nitrogen, iron and manganese metabolisms are shown in Figure 8A. A representative example is shown by the nitrate/nitrite transporter, *narK*, involved in nitrate assimilation, which was present in all 11 metagenomes (Figure 8A) but was not significantly correlated (correlation coefficient of 0.26) with the sediment nitrate concentrations (2.5–8.1 ppm, Table 1). Two gene categories corresponding to (i) nitrogen fixation (*nif*, nitrogenase, EC:1.18.6.1) and (ii) dissimilatory nitrate reduction to ammonia (DNRA) (*nrfA*, nitrite reductase cytochrome c-552, EC:1.7.2.2) were enriched 3–5 fold in cluster B metagenomes, compared with the others. Other genes involved in nitrogen cycling that were abundant included (i) *nirB* and *nirD* nitrite reductases (NADH) (EC:1.7.1.15, K00362, K00363), *nirK* NO-forming (EC:1.7.2.1, K00368) and (ii) *norB* nitric-oxide reductase (cytochrome c) (EC:1.7.2.5, K04561) (Figure 8A). The corresponding enrichment patterns were not significantly correlated with each other nor with the nutrient concentrations in the samples. These patterns were also not associated with specific metagenome clusters from Figure 5 (data not shown).

A significant correlation (*p* ≤ 0.05) was found between relative gene abundance and the sediment bulk chemistry for the manganese transport protein *mntH* (KO:K03322) and Mn concentrations (ppm) (Figures 8B,C). Enrichment of *mntH* was also significantly correlated (*p* ≤ 0.001) with the abundance of potassium uptake-related genes: *kup* (KO:K03549) (Figure 7B) and an osmosensitive K^+^ channel with an histidine kinase sensor domain (PF02702), which are specialized transporters for potassium uptake against very high transmembrane concentration gradients in freshwater and low-salinity environments (Banuelos et al., 1995). In contrast, the higher salinity Baker Bay metagenomes were enriched with *kefA*, the gene encoding a potassium efflux protein (KO:K05802) involved in salinity adaptation (Figure 8B). The Baker Bay metagenomes of cluster A were also enriched with several other gene categories associated with salinity adaptations and protection from osmotic stress. These gene categories included glycine betaine/proline transport system, *proV-W* (KO:K02000-2001) (Figure 8B), Na+:H+ antiporter of the NhaB family (KO:K03314), and ectoine hydrolase *doeA* (KO:K15783), all of which are involved in adaptation of bacteria to halophilic environments (Widderich et al., 2016).

### Eukaryl Community Composition

The majority (>68%) of Eukaryota sequences detected at ≥30% identity level across all sediment metagenomes represented diatoms (Bacillariophyta) that were associated with either the class Bacillariophyceae or Coscinodiscophyceae (Table 2). The next most abundant sequences identified (1–2%) were affiliated with taxa from Annelida, Arthropoda, Ascomycota, Chlorophyta, Chordata, Mollusca, Streptophyta and unclassified phyla.

**TABLE 2:** Abundances of eukaryotic and viral taxa compared to the Bacillariophyta abundance in the sediment metagenomes.

**Taxon^a^**	**S.41_**	**S.184_**	**S.182_**	**S.186_**	**S.180_**	**S.43_**	**S.178_**	**S.42_**	**S.174_**	**S.190_**	**S.188_**	***R*^2b^**
	**BBB**	**BBB**	**BBI**	**BBA**	**BBC**	**YBM**	**YBM**	**YBB**	**CBK**	**CBC**	**CBA**	
Bacillariophyta	0.01	0.56	15.49	26.06	14.78	0.15	3.1	0.01	0.28	0.18	0.22	1
Phycodnaviridae	0	0.02	0.09	0.13	0.03	0.01	0.01	0.01	0	0	0.02	0.94
Heterolobosea	0.01	0	0.03	0.06	0.03	0	0.01	0	0	0	0.01	0.99
Oligohymenophorea	0.02	0.01	0.04	0.07	0.04	0.01	0.02	0.01	0.01	0.01	0.01	0.97
Choanoflagellida	0.02	0.02	0.11	0.16	0.09	0.01	0.05	0.02	0.01	0.01	0.02	0.99
Branchiopoda	0	0.02	0.09	0.13	0.05	0.03	0.05	0.01	0.01	0.01	0.01	0.95
Gastropoda	0.01	0.03	0.06	0.06	0.04	0.04	0.37	0.06	0.01	0.01	0.01	0.05

*^*a*^Percentage of the total number of CDS with taxonomic annotations ≥ 30% identity ≥ 70% of the alignment length. ^*b*^Pairwise correlation coefficients shown in the last column were calculated between the abundance values of each taxon (shown in the corresponding row) and the Bacillariophyta data (the top row).*

### Phylogenetic Analysis of Phytoplankton in the Sediment Metagenomes

To identify diatom taxa that were deposited in the lateral bay sediments, we analyzed the *rbcL* gene encoding the large subunit of ribulose-bisphosphate carboxylase (RuBisCO, EC 4.1.1.39), which is widely used for diatom classification (Evans et al., 2008). Overall, the corresponding KEGG Orthology (KO, Kanehisa et al., 2011)^4^ category KO:K01601 contained 1309 predicted genes from the 11 sediment metagenomes. The relative abundance of diatom *rbcL* genes in individual metagenomes ranged from 0.8 to 80%, with the highest values observed in the cluster A metagenomes from Baker Bay. Other identified eukaryotic *rbcL* genes corresponded to Chlorophyta (as high as 8 and 11% in S.188_CBA and S.174_CBK, respectively), and Charophyta (1.5% in S.188_CBA). Archaeal and bacterial genes encoding RuBisCO were also identified (data not shown).

**FIGURE 9:**
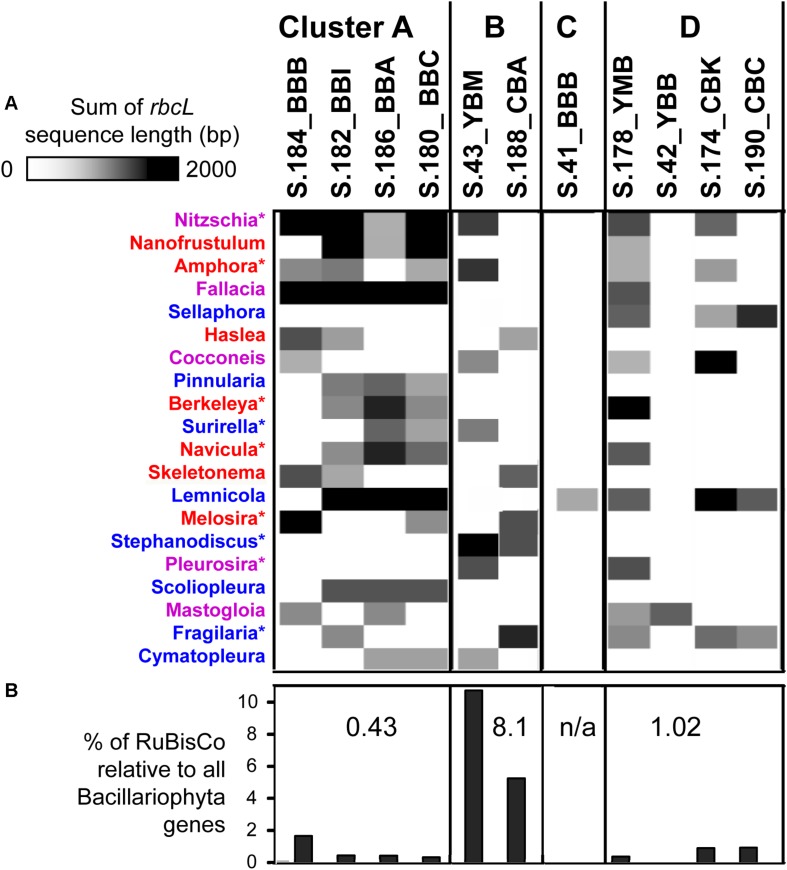
**(A)** 1D hierarchical clustering (correlation uncentered, average linkage) performed to identify co-occurring families of diatoms across metagenomes. The heat map shows phylogenetic composition of Bacillariophyta genes from the functional gene category KO:K01601 (EC 4.1.1.39), ribulose-bisphosphate carboxylase (RuBisCO). In depth annotations were generated for RuBisCo genes ≥ 500 bp length. Colors indicate the diatom genus origin: blue, freshwater; red, marine; purple, both habitats. ^∗^Indicate the genera (9 out of 20) that were observed microscopically in the Columbia River lateral bays previously (Simenstad et al., 1984). **(B)** Percentages of RuBisCO genes relative to all predicted Bacillariophyta genes with ENZYME (EC) annotations. Numbers indicate median percentage values for each cluster. The metagenomes are grouped into the four clusters (A, B, C, and D) corresponding to those shown in Figure 5.

Further analysis of diatom *rbcL* was carried out for predicted genes of length ≥500 bp (>1/3 of the full-length *rbcL*). In-depth annotations revealed high identities to sequenced references (minimum sequence identity of 95%, with an average of 97%, calculated for the entire alignment length). Approximately 20 different diatom genera, most of them pelagic, were identified as being present in at least two metagenomes (Figure 9A). The highest diversity was observed in cluster A metagenomes, each containing a mixture of 8–12 different diatom *rbcL* genes. Although the relative abundance of diatom sequences was much lower in the S.184_BBB metagenome from cluster A (0.5% compared to 26% in S.186_BBA), analysis of *rbcL* genes revealed the presence of at least 8 diatom genera in that sample, most of which overlapped with the other cluster A metagenomes. These genes were annotated to marine, freshwater, and brackish diatom genera with both planktonic and benthic representatives. Cluster B metagenomes showed less variation in diatom genera (7 and 5 in S.43_YBM and S.188_CBA, respectively) sharing only one common genus. Large differences were also observed for cluster D, ranging from 1 to 12 genera with some overlap among the metagenomes. Two metagenomes, S.42 YBB and S.41_BBB, contained only a single diatom genus, the brackish benthic diatom *Mastogloia* and the freshwater diatom *Lemnicola*, respectively (Figure 9A).

### Living or Dead Diatoms Inferred From RuBisCO:Nuclear Gene Ratio Proxy

The three cluster A metagenomes containing a high relative abundance of diatom genes (Table 1) had a relatively low percentage of RuBisCO genes (0.32–0.42%, Figure 9B). In contrast, cluster B metagenomes contained a relatively low overall abundance of diatom genes (Table 1) but a high percentage of RuBisCO sequences (5-10%, enriched 15-25X compared to all other metagenomes). The three metagenomes belonging to cluster D also contained a low relative abundance of diatom genes, ranging from 0.5 to 1% RuBisCO. Taken together, there was a significantly lower ratio of chloroplast-encoded RuBisCO:nuclear genes in clusters A and D, indicating a higher percentage of living diatoms in those samples. This was in comparison to cluster B, which had a higher ratio of chloroplast-encoded RuBisCO:nuclear genes, indicating a higher proportion of dead diatoms with more highly degraded nuclear DNA (Figure 9B).

### Predators and Grazers Associated With Diatoms in Cluster A Metagenomes

The Bacillariophyta enrichment in cluster A metagenomes was also correlated (*p* ≤ 0.001, Table 2) to high relative abundance of predators and grazers. These organisms included the bacterivores Heterolobosea and Choanoflagellida, the microphagous heterotrophic ciliate Oligohymenophorea, and the crustacean predator copepod Branchiopoda (Table 2). The enrichment patterns of other Eukaryota were not correlated with Bacillariophyta. For example, no correlation was observed for an invasive New Zealand Gastropoda, *Potamopyrgus antipodarum. P. antipodarum* is frequently observed in summer in the Columbia River estuary (Bersine et al., 2008) and was present at low abundance in most metagenomes, while highly enriched in the S.178_YBM sample (Table 2). Other Eukaryota, mostly marine in origin, such as Cnidaria (polyps) and Porifera (sponges) were enriched in the sediment metagenomes according to the degree of marine influence, with near absence in freshwater locations and higher abundance in both Baker Bay and Youngs Bay mouth (data not shown).

### Viruses and Bacteriophage

**FIGURE 10:**
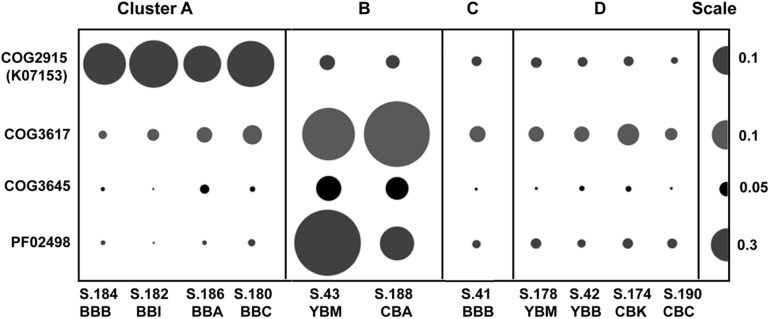
Abundances of functional gene categories involved in regulation of bacteriophage life cycle (lysogenization and lytic induction). COG2915, negative regulator of phage lambda lysogenization *hflD* encoded in bacterial genomes; COG3617, COG3645, PF02498, phage/prophage antirepressor genes encoded by bacteriophage genomes. Abundance values are proportionate to the bubble width. The scale for each row is indicated with a half-bubble to the right, and its diameter corresponds to genes/genome values indicated by a number. The metagenomes are grouped into four clusters (A, B, C, and D) corresponding to those shown in Figure 5.

Two of the metagenome clusters were notable with respect to virus/phage sequences. Cluster A metagenomes contained a high relative abundance of viral sequences compared to all other metagenomes (with an average of 0.55 vs. 0.14%, Table 1). We observed two different abundance patterns for the predominant viral taxa. Abundance of Phycodnaviridae was significantly correlated (*p* ≤ 0.001) with diatom abundance (Table 2). Enrichment of tailed bacteriophages of the order Caudovirales was significantly correlated (*p* < 0.02) with abundance of several Bacteroidetes families (Figure 6A). Cluster A metagenomes were enriched not only with phage genes, but also with the bacterial host factor *hflD* (Figure 10), which is involved in resistance to phage infection and regulation of lysogeny in response to the ratio of phage to host cells (Avlund et al., 2009).

Cluster B metagenomes also contained higher relative abundance of bacteriophage genes (0.13–0.23%) compared to clusters C and D, but this was still 2–3 times lower compared to cluster A (Figure 6). However, both of the cluster B metagenomes showed high enrichment (at least 5X greater than all other metagenomes) of three specific phage-encoded genes annotated as phage/prophage antirepressors (Lemire et al., 2011) (Figure 10).

## Discussion

### Sediment Metagenomes Cluster by Water Salinity and Nutritional Status

Our metagenome analyses indicated that the taxonomic and metabolic composition of archaeal and bacterial communities in Columbia River estuarine sediments were influenced primarily by: (1) water salinity and (2) access to labile organic matter in the form of diatom detritus from allochthonous blooms. Research in the Columbia River estuarine water column (Fortunato et al., 2013; Fortunato and Crump, 2015) revealed the importance of water salinity as a major driver of taxonomic diversity for bacterial and archaeal populations and we anticipated that the same would be true for shaping microbial communities in estuarine sediments. Our results, however, also suggest that nutritional status was an overriding determinant of similarity among the sediment metagenomes. Our data showed that metagenomes from the freshwater Cathlamet and brackish water Youngs Bay sediments grouped together into clusters B and D, distinguished by high or low sequence abundance, respectively, of bloom-associated Bacteroidetes. Furthermore, metagenomes from sediments of the higher-salinity Baker Bay were split into two clusters, A and C, distinguished by the presence or absence, respectively, of abundant sequences of diatoms, bloom-associated bacteria (Bacteroidetes and Alteromonadaceae) and viruses known to infect algal and Bacteroidetes taxa.

### Physiological State of Diatoms Influences Sediment Metagenome Clusters

We sought to better understand the differences between clusters A and B, which both contained abundant bloom-degrading bacterial taxa but generally differed in relative abundance of diatom sequences. While the proportion of living vs. dead phytoplankton can be inferred using the ratio between chl *a* and particulate organic carbon (POC) in water samples (Prahl et al., 1997), this approach is not applicable to sediments because they contain relatively large quantities of recalcitrant organic carbon and low chl *a* concentrations (Daemen, 1986). In previous work, we observed the dramatic enrichment of chloroplast over nuclear-encoded genetic material in samples containing biogeochemical and functional gene indicators for the advanced stages of diatom bloom degradation (Smith et al., 2013, 2015). This finding is consistent with the idea that upon cell death, diatom chloroplast DNA persists in the environment much longer than nuclear DNA because of the extra protection afforded by having four membranes, in contrast to the dual membrane-bound nucleus (Lang et al., 1998). Thus, we hypothesized that the ratio between chloroplast and nuclear DNA (the latter encoding > 98% of all diatom proteins) (Armbrust et al., 2004; Lopez et al., 2005) in environmental samples may serve as a proxy for the general physiological state of diatoms. To test this, we calculated the proportion of diatom chloroplast-encoded *rbcL* genes encoding the large subunit of RuBisCO relative to total nuclear DNA gene sequences (Smith et al., 2013, 2015). Although this hypothesis remains to be rigorously tested, our results (Figure 9B) indicated that the inferred physiological status of diatoms (higher proportion of intact cells vs. higher proportion of degraded cells) calculated from the *rbcL* gene:nuclear gene abundance ratios in our metagenomes is consistent with the abundance values observed for genes involved in bacterial uptake of compounds expected to occur in early vs. later-stage bloom degradation. It is furthermore important to note that the wide variation in gene copy numbers observed in algal nuclear and chloroplast genomes (Armbrust et al., 2004; Bedoshvili et al., 2009; D’Alelio et al., 2009) precludes the use of 16S (chloroplast) to 18S (nuclear) rRNA gene ratios for this purpose.

Early stages of diatom bloom degradation in the 2013 Baker Bay sediments are indicated in the cluster A metagenomes by the low percentage of diatom RuBisCO compared to nuclear-encoded diatom genes (Figure 9B), suggesting relatively high abundance of whole diatom cells rather than cell-free chloroplasts. Blooms occurring near the time of sampling (Figure 2B) represented a major influx of pelagic phytoplankton, followed by sinking and deposition of non-degraded diatoms. This influx was associated with the relatively abundant sequences of bloom-utilizing bacteria (Bacteroidetes and Alteromonadaceae) (Figure 6A), carrying functional genes involved in the uptake of labile, phytoplankton-derived DOC, including transporters for low molecular weight compounds such as amino acids and sugars (Figure 7). Other bacterial functional gene categories enriched in these metagenomes, such as *chpC* (He and Bauer, 2014) and *folM* (Feirer et al., 2015), are involved in locating and colonizing detrital particles, respectively (Figure 7). This is consistent with the idea that particle colonization by free-living bacteria occurs during the early stages of diatom bloom utilization (Riemann and Grossart, 2008).

Cluster B metagenomes showed extreme enrichment of the chloroplast-encoded RuBisCO gene *rbcL* (Figure 9B). According to our working hypothesis (Smith et al., 2015, 2017), poor preservation of nuclear DNA together with the simultaneous persistence of chloroplast genes indicates relatively late stages of diatom bloom degradation. Supporting this idea, the Bacteroidetes taxa most highly enriched in cluster B (Figure 6B) are largely distinct from those in cluster A (Figure 6A). The cluster B metagenomes are also enriched for metabolic genes involved in the later stages of phytoplankton bloom degradation (Figure 7), once the freshly produced DOC has been utilized and only high molecular weight polymers of phytoplankton-derived POM remain (Teeling et al., 2012; Klindworth et al., 2014). Large suites of genes involved in degradation of cell-wall polysaccharides and utilization of complex organic matter are known to be present in the corresponding genomes of, in particular, Flavobacteria (Gonzalez et al., 2011; Gómez-Pereira et al., 2012; Fernandez-Gomez et al., 2013).

### Phylogenetic and Sensor Data Indicate Multiple Blooms Deposited Diatoms in Sediments

In marine habitats, the bacterial community composition is profoundly affected by blooming phytoplankton – the turnover of which provides an important source of organic carbon for the entire food web, including bacterial heterotrophs, protist grazers, other zooplankton, and benthic consumers (Frey et al., 1984; Small et al., 1990; Cloern, 1996). Periodic accumulation of phytoplankton biomass is a typical feature of shallow coastal ecosystems including bays, estuaries, and tidal rivers (Cloern, 1996). These blooms are typically dominated by a few different species (Verity and Smetacek, 1996; Smith et al., 2012). Given that our *rbcL* data indicated the presence of up to 12 different diatom genera of marine, brackish and freshwater origin within the same sediment metagenomes (Figure 8A), multiple bloom deposition events likely occurred at the majority of sampling locations. Consistent with this, sensor data showed multiple, pronounced peaks of chl *a* in the estuarine water column for 4 weeks prior to sampling (Figure 2B). The majority of diatoms shared high identity with pelagic genera of marine and riverine origin, and therefore most likely represented allochthonous blooms that were transported from the main estuarine channels into the lateral bays and deposited in sediments.

### Inferred Phage Dynamics

In coastal areas and seawater microcosms, lytic phage production has been correlated with bacterial propagation and particle colonization during phytoplankton blooms (Steward et al., 1992; Riemann and Grossart, 2008; Knowles et al., 2016). Evidence for lytic phage infection in the early stages of bloom degradation is observed in cluster A sediment metagenomes. In particular, metagenome S.186_BBA in cluster A has the following features: (i) high relative abundance of diatom sequences (26%), (ii) low relative abundance of Bacteroidetes sequences (9%), and (iii) high relative abundance of bacteriophage genes (0.46%), compared to metagenomes in clusters B, C, and D (Table 1 and Figure 6A). The predominantly lytic propagation of phages is suggested (Wegrzyn and Wegrzyn, 2005) for cluster A metagenomes by the relatively high abundance of the bacterial *hflD* gene (COG2915, Figure 10), a negative regulator of lysogenization (Kihara et al., 2001). Nutrient fluxes resulting from phytoplankton and bacterioplankton lysis typically attract grazers and predators (Smith et al., 2017), which were also observed in comparatively high abundance in the four Baker Bay sediment metagenomes forming cluster A (Table 2).

In contrast, the bacteria in cluster B metagenomes contained a lower abundance of bacteriophage genes (Table 1 and Figure 6A). Massive phage infection during early diatom bloom utilization (as proposed for cluster A) may result in high selective pressure and an increase in host resistance, with a subsequent decrease in lysogenic phage content. In addition, co-evolution of bacterial hosts and phages upon recurring infections can result in emergence of phages capable of coordinated survival during host lysis (Labrie et al., 2010; Rodriguez-Brito et al., 2010; Hall et al., 2011; Weitz et al., 2013). A common mechanism associated with phage ‘antirepressor’ genes ensures synchronous induction of multiple prophages and thus, concerted phage survival upon death of a polylysogenic bacterial host (Wegrzyn and Wegrzyn, 2005; Lemire et al., 2011). Phage antirepressor genes are highly enriched in cluster B metagenomes (Figure 10), suggesting that the corresponding microbial communities may have undergone positive selection for this phage genome feature in the past. Since this selection occurs during the early stages of diatom bloom utilization, the cluster B metagenomes therefore represent the later stages of this process.

Our prior data (Smith et al., 2017) from samples collected in the Columbia River estuarine water column suggested that bacteriophages switch from a lysogenic to lytic life cycle upon rapid propagation of bacterial hosts engaged in early stages of diatom bloom degradation. Referred to as ‘Kill-the-Winner,’ this strategy involves an increase in lytic infection with increase in host density, and is believed to be important for maintenance of host diversity through suppression of rapidly propagating strains (for a review see Knowles et al., 2016). Such lytic phage attack may result in a dramatic decrease in bacterial host abundance during the initial stages of POM colonization (Riemann and Grossart, 2008), and provide both dissolved and particulate organic matter to the community of free-living microorganisms through the “viral shunt” (Fuhrman, 1999; Suttle, 2005; Buchan et al., 2014). We propose this scenario may have occurred in the S.186_BBA sediments around the time of sampling, given the relatively high abundance of diatom and phage sequences combined with the relatively low abundance of bloom-utilizing bacteria (Figure 6 and Table 2).

### Metagenome Profiles in the Absence of Labile Organic Matter

The low relative abundance of diatom genes in cluster C and D metagenomes indicated the absence of substantial decaying phytoplankton detritus, and is consistent with a low abundance of identified bloom-utilizing bacteria (Figure 6). Taxonomic profiles of C and D clusters were characterized by an increased relative abundance of chemolithotrophs. These clusters also revealed an overall functional complement enriched for genes involved in degradation and uptake of recalcitrant DOC and general nutrient scavenging (Figure 7). A report from the South China Sea indicates that nutrient scavenging plays an important role not only in oligotrophic marine environments, but also in productive coastal ecosystems in the absence of fresh phytoplankton inputs (Dong et al., 2014). Furthermore, the enzyme CODH, highly enriched in these metagenomes, catalyzes reduction of CO_2_ to CO (Ragsdale and Wood, 1991) and aerobic and anaerobic CO oxidation (King and Weber, 2007) in pathways spanning a variety of lithotrophic metabolisms.

Two metagenomes, S.178_YBM and S.184_BBB, do not fit the scenarios discussed above very well. The relative abundance of diatom genes was 3 and 0.6%, respectively (Table 1) and the presence of multiple and diverse diatom *rbcL* (Figure 9) suggested mixing of material from multiple bloom deposition events. Notably, the enrichment (3%) of diatom genes observed in S.178_YBM was not accompanied by enrichment of known bloom-utilizing bacteria (Figure 6) or the corresponding functional gene profile. This metagenome clustered together with three metagenomes that did not contain diatom genes (Figure 5). Thus, despite the diatom presence, the S.178_YBM and S.184_BBB microbial communities appeared to have returned to a ‘pre-bloom’ composition and metabolic state. We speculate that these samples likely contained quiescent diatom cells, probably from earlier bloom events. Generally, unfavorable conditions lead to diatom aggregation and sinking, facilitating survival of a small proportion of cells in resting state (or as spores) in dark bottom waters (Smetacek, 1985; Verity and Smetacek, 1996; Itakura et al., 1997). Previous studies showed that in shallow estuarine and tidal flat sediments, some diatoms may remain alive (with a maximum survival rate of 30 and 10% after 3 months and 1 year, respectively), whereas the rest of deposited cells die and are quickly degraded by heterotrophic bacteria (Veuger and van Oevelen, 2011).

### Sequence Markers of Salinity

Analysis of metagenome functional gene content also corroborated the influence of salinity variation on sediment microbial populations. Two genes involved in active uptake of potassium, *kup* (KO:K03549, Figure 6B) and an unnamed osmosensitive K^+^ channel with a histidine kinase sensor domain (PF02702), were relatively more abundant in Youngs and Cathlamet Bay sediment metagenomes associated with lower water salinities. Conversely, the Baker Bay metagenomes were enriched with several gene categories associated with salinity adaptations and protection from osmotic stress (Figure 6B), including the *kefA* (KO:K05802) gene coding for a potassium efflux protein, the glycine betaine/proline transport system, *proV-W* (KO:K02000-2001), an Na+:H+ antiporter of the NhaB family (KO:K03314), and the *doeA* gene (KO:K15783) for ectoine hydrolase. The *kefA* enrichment pattern was significantly negatively correlated (*p* ≤ 0.001) to *kup*. Since the former functions in potassium uptake under freshwater conditions, and the latter in potassium efflux in saline ocean water, these gene sequences may also serve as salinity markers indicating the prevailing conditions in the bay sediments around the sampling locations.

### Influence of River and Ocean End Members on Archaeal Taxa in Lateral Bay Sediments

Marine Archaea were predominant at the Baker Bay sites that are strongly influenced by oceanic salinity intrusion, while soil Archaea (likely transported by river water) were more prevalent at the lower-salinity and freshwater sites of Cathlamet Bay and the Youngs Bay back region. Thus, the Nitrosopumilaceae enrichment observed in the Baker Bay metagenomes may be due to the Bay’s proximity to the Columbia River mouth and consequent marine influence.

### Dominant Bacterial Taxa Involved in Diatom Degradation in Lateral Bay Sediments

Together with other studies (Smith et al., 2015, 2017) our data show that Bacteroidetes and Gammaproteobacteria (Alteromonadaceae) taxa are major players in diatom bloom consumption in Columbia River estuarine sediments and the water column. A cosmopolitan, taxonomically diverse, and physiologically broad group of heterotrophs observed in marine, freshwater, sediment (including tidal salt marshes) and soil habitats, Bacteroidetes typically constitute from 5 to over 20% of all bacteria (Kirchman, 2002; Lydell et al., 2004; Fernandez-Gomez et al., 2013). Within Bacteroidetes the Flavobacteriia class, in particular, is consistently associated with phytoplankton blooms, and has been implicated in biopolymer degradation and utilization (Buchan et al., 2014). In this study, taxa in the classes Flavobacteriia and Cytophagia were generally abundant in sediments across the different stages of phytoplankton utilization. However, taxa from the class Bacteroidia and certain members of the Sphingobacteriia class were relatively more abundant during the later stages of diatom bloom degradation (Figure 6). Although taxa from the Bacteroidia class are not generally as well-characterized in environmental studies, they have major roles in the breakdown of dietary polysaccharides as abundant members of mammalian intestinal microbiota (Thomas et al., 2011).

While members of the Alteromonaceae have been identified as phytoplankton bloom-associated taxa, relatively little physiological and genomic information has been uncovered related to their specific roles (Tada et al., 2011; Buchan et al., 2014). This largely marine taxon consists of obligately aerobic heterotrophs capable of extensive degradation of a variety of substrates (López-Pérez and Rodriguez-Valera, 2014). In this study on estuarine sediments, Alteromonaceae appeared to be abundant only during the initial stages of phytoplankton utilization.

## Conclusion

Our research revealed that in a fast-flowing estuary access to diatom detritus is a critical factor affecting sedimentary bacterial community composition and metabolic potential. Somewhat unexpectedly, diatom presence in sediments partially superseded the influence of water salinity and geographic location, resulting in metagenomes from disparate bays grouping together by nutritional status in our cluster analysis. These results highlight a central role for allochthonous labile organic matter (i.e., diatom detritus), in shaping bacterial taxonomic and functional properties in Columbia River estuary lateral bay sediments. They further suggest that in river-dominated estuaries, sediment microbial communities in areas of extended water retention may contribute disproportionately to estuarine organic matter degradation and recycling. The functional roles of key taxa, such as Bacteroidetes and Alteromonadaceae, in organic matter turnover in the Columbia River estuarine water column (Smith et al., 2017) and sediments (Smith et al., 2015) need to be accounted for in nutrient cycling models (Baptista et al., 2015). Doing so will help elucidate overlooked contributions of major sedimentary heterotrophs in the biogeochemistry of fast-flowing estuaries, which serve as dynamic interfaces between terrestrial and oceanic ecosystems around the world.

## Data Availability Statement

The datasets generated for this study can be found in the IMG/M-ER metagenome database, designated with GOLD ID Gs0047387 (Marine and estuarine microbial communities from Columbia River Coastal Margin). IMG/M-ER accession numbers: taxonoid (taxon object identification): 3300005832, 3300005825, 3300005828, 3300005826, 3300005824, 3300005831, 3300005830, 3300005836, 3300005827, 3300005829, 3300005833.

## Author Contributions

MS, HS, and LH designed the study. MS, LH, and HS collected and/or processed the sediment samples for DNA sequencing. MS analyzed the sequence data and performed the statistical analyses. AR performed the sequence assembly. MS and HS wrote the manuscript with contributions from LH and AR. All authors reviewed and approved the manuscript.

## Conflict of Interest

The authors declare that the research was conducted in the absence of any commercial or financial relationships that could be construed as a potential conflict of interest.

## Footnotes

1 http://www.stccmop.org/datamart/observation_network

2 https://sourceforge.net/projects/bbmap/

3 https://img.jgi.doe.gov/cgi-bin/mer/main.cgi

4 http://www.genome.jp/kegg/ko.html
